# NAD^+^ precursors promote the restoration of spermatogenesis in busulfan-treated mice through inhibiting Sirt2-regulated ferroptosis

**DOI:** 10.7150/thno.92416

**Published:** 2024-04-15

**Authors:** Yan-Qin Feng, Xuan Liu, Ning Zuo, Mu-Bin Yu, Wen-Meng Bian, Bao-Quan Han, Zhong-Yi Sun, Massimo De Felici, Wei Shen, Lan Li

**Affiliations:** 1 College of Life Sciences, Key Laboratory of Animal Reproduction and Biotechnology in Universities of Shandong, Qingdao Agricultural University, Qingdao 266109, China.; 2 Department of Urology, Shenzhen University General Hospital, Shenzhen 518055, China.; 3 Department of Biomedicine and Prevention, University of Rome Tor Vergata, Rome 00133, Italy.

**Keywords:** NAD^+^ precursors, busulfan, spermatogenesis, ferroptosis, SIRT2

## Abstract

**Rationale:** In recent years, nicotinamide adenine dinucleotide (NAD^+^) precursors (Npre) have been widely employed to ameliorate female reproductive problems in both humans and animal models. However, whether and how Npre plays a role in the male reproductive disorder has not been fully clarified.

**Methods:** In the present study, a busulfan-induced non-obstructive azoospermic mouse model was used, and Npre was administered for five weeks following the drug injection, with the objective of reinstating spermatogenesis and fertility. Initially, we assessed the NAD^+^ level, germ cell types, semen parameters and sperm fertilization capability. Subsequently, testis tissues were examined through RNA sequencing analysis, ELISA, H&E, immunofluorescence, quantitative real-time PCR, and Western blotting techniques.

**Results:** The results indicated that Npre restored normal level of NAD^+^ in blood and significantly alleviated the deleterious effects of busulfan (BU) on spermatogenesis, thereby partially reestablishing fertilization capacity. Transcriptome analysis, along with recovery of testicular Fe^2+^, GSH, NADPH, and MDA levels, impaired by BU, and the fact that Fer-1, an inhibitor of ferroptosis, restored spermatogenesis and semen parameters close to CTRL values, supported such possibility. Interestingly, the reduction in SIRT2 protein level by the specific inhibitor AGK2 attenuated the beneficial effects of Npre on spermatogenesis and ferroptosis by affecting PGC-1α and ACLY protein levels, thus suggesting how these compounds might confer spermatogenesis protection.

**Conclusion:** Collectively, these findings indicate that NAD^+^ protects spermatogenesis against ferroptosis, probably through SIRT2 dependent mechanisms. This underscores the considerable potential of Npre supplementation as a feasible strategy for preserving or restoring spermatogenesis in specific conditions of male infertility and as adjuvant therapy to preserve male fertility in cancer patients receiving sterilizing treatments.

## Introduction

In recent years, male infertility rate has risen at an alarming rate globally 1. The main causes of such condition are disorders of spermatogenesis 2, 3. In adults, this process comprises subsequent complex phases. These phases include the mitosis of spermatogonia stem cells (SSCs), the SSCs self-renew, and their maturation into differentiating mitotic spermatogonia (SPGs), Subsequently, SPGs enter meiosis as spermatocytes I (SPCs I), undergo the first meiotic division to become spermatocytes II (SPCs II), undergo the second meiotic division to produce haploid spermatids (STs), and finally, STs mature into spermatozoa 4. Disturbances in any of these processes can easily lead to reduced fertility or infertility. However, the underlying mechanisms and therapeutic strategies applicable in such conditions often remain elusive.

Busulfan (1, 4-butanediol dimethanesulfonate) (BU) is commonly employed to induce prolonged azoospermia in experimental animals. It shows cytotoxic effects by forming DNA-DNA cross-links, DNA-protein cross-links, and single strand breaks. In the adult testis, BU primarily exerts its toxic effects on cells in the G1 phase, it kills mainly mitotic spermatogonia through apoptosis, autophagy and ferroptosis leading to infertility 5-8. Growth factors such as glial-derived neurotrophic factor (GDNF), liver growth factor (LGF), and substance P (SP) have demonstrated the ability to partially restore spermatogenesis impaired by BU, likely by favoring the reactivation of the SPGs compartment in the seminiferous epithelium 9-11. Relevant for the present study, melatonin and alginate oligosaccharides improved spermatogenesis in infertile BU treated mice through the reduction of testicular reactive oxygen species (ROS) production 5 or meliorating general metabolism 12. Furthermore, vitamin A precursor supplementation likely through the antiapoptotic and antioxidant activities of its active metabolite ATRA (all-trans retinoic acid), has been proved to ameliorate male reproductive disorders in mice induced by BU treatment 13. Additionally, supporting the role of impaired metabolism and ROS in male infertility, clinical studies have reported that 30% of such conditions are associated with low testicular level of glutathione peroxidase 4 (GPX4), a unique intracellular antioxidant enzyme that can directly reduce lipid peroxidation and inhibit ferroptosis outcomes 14.

Nicotinamide adenine dinucleotide (NAD^+^) is an essential cofactor present in the cytosol, nucleus, and mitochondria, capable of regulating cell redox homeostasis and, as such, crucial for cellular metabolism 15, 16. Within the body, NAD^+^ biosynthetic pathways include the kynurenine pathway, which utilizes L-tryptophan, the Preiss-Handler pathway synthesized from Nicotinic acid (NA), and the salvage pathway, which generates NAD^+^ through Niacinamide (NAM) 16. Several studies have reported that the addition of NAD^+^ precursors, such as NA, NAM, Nicotinamide mononucleotide (NMN), and Nicotinamide riboside (NR), can efficiently elevate blood and tissue levels of NAD^+^ 17-21. In reproductive tissues, a high level of NAD^+^ in the ovary has been associated with improved female fertility 22, 23. Interestingly, studies have shown that *in vivo* supplementation of the NMN and NA effectively improves the quality of maternally aged oocytes by restoring their mitochondrial function and enhancing meiotic competency, fertilization ability, and subsequent embryonic development potential 24, 25. Additionally, NA supplementation has been found to reverse meiotic defects and metabolic disorders in the oocytes of obese mice 26. Consistent with these findings, Min et al. demonstrated that NAM supplementation improves oocyte quality and offspring development in aged *Caenorhabditis elegans* by alleviating mitochondrial dysfunction 21.

Physiological effects of low NAD^+^ status and their potential impact on male fertility have been difficult to study due to a lack of suitable animal models. However, in recent studies, NAD^+^ levels have been found associated with a decline of spermatogenesis in a transgenic mouse model and aging mice 27.

In the present study, we established an infertility mouse model with a single intraperitoneal injection of BU and supplemented the mice with NAD^+^ precursors (Npre) to explore whether they alleviate spermatogenesis disorders and infertility caused by the drug. Based on the outcome, we hypothesized that NAD^+^ exerts protective effects on spermatogenesis dysfunctions by supporting the anti-ferroptosis effects of sirtuins, particularly SIRT2. Thus, we propose Npre supplementation as a novel strategy to ameliorate some conditions of male infertility including cancer therapy using alkylating compound such as BU.

## Results

### Npre improves spermatogenesis in busulfan-treated mice

**Figure 1:**
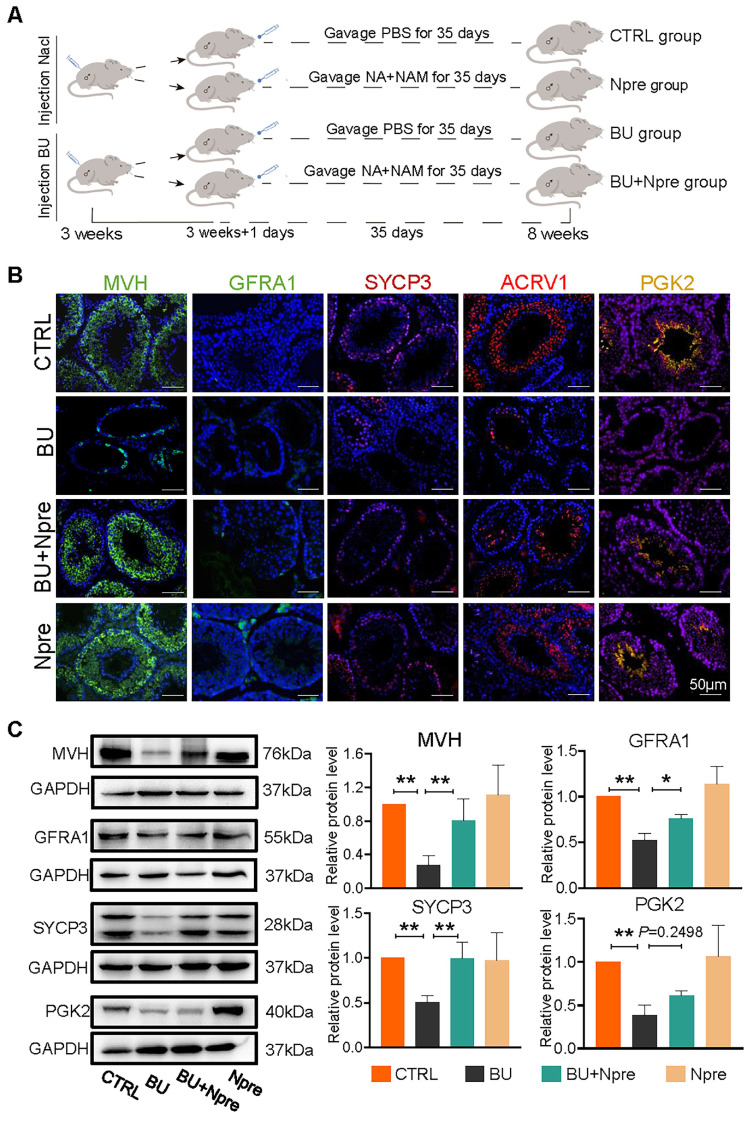
**Npre improves spermatogenesis in BU-treated mice.** Experimental design diagram. (B) Representative IF images for MVH (germ cells), GFRA1 (SSCs), SYCP3 (SPCs), ACRV1 and PGK2 (spermatozoa) in tissue sections of seminiferous tubules of testes of the indicated groups; The nucleus was stained with Hoechst 33342. Scale bar=50 μm. (C) Left, representative WB images of MVH, GFRA1, SYCP3 and PGK2 of protein extracts from testes of the same mice groups in B; right, quantification of the protein levels of the same protein of B relative to GAPDH used as control. Testis samples were taken from at least 3 to 6 mice in each group and data from at least three independent replicates are showed as mean ± SD (^*^P < 0.05; ^**^P < 0.01, ns=not significant).

Having established a model of reversible infertility in male mice with an injection of 20 mg/kg BU 28, we assessed whether supplementation with Npre for five weeks following BU injection could restore spermatogenesis and improve sperm quality (Figure 1A).

In preliminary experiments, concentrations of 10 mg/kg, 50 mg/kg, 100 mg/kg, 200 mg/kg and 400 mg/kg of Npre were dispensed. The results showed a comparable, progressive increase in the body weights of both CTRL and treated mice across all Npre concentration (Figure S1A). However, BU administration led to a significant reduction in NAD^+^ concentration in blood, whereas supplementation with Npre in the range of 50-400 mg/kg restored NAD^+^ concentrations to levels comparable to those of the CTRL group (Figure S1B). Likewise, the reduction in seminiferous tubule surface area and alterations in blood content levels of inhibin B (INH-B), follicle-stimulating hormone (FSH), and testosterone (T) caused by BU treatment were also significantly alleviated by 200 and 400 mg/kg of Npre (Figure S1C-D). To ensure the safety of Npre in mice, the pro-inflammatory cytokines were detected in the blood after 35 days of Npre treatment, revealing no significant difference in IL-6 and IL-10 levels between Npre with CTRL groups (Figure S2A). Furthermore, the livers, intestines, and ovaries of mice in the Npre group were examined, with no abnormalities observed found in tissue section (Figure S2B). Following such results, 200 mg/kg of Npre was selected for use in all subsequent experiments.

We next investigated whether the Npre effect on the seminiferous tubule impinged on particular germ cell types. Immunofluorescence (IF) staining of marker proteins of germ cells MVH, GFRA1 for SSCs, SYCP3 for SPCs, ACRV1 for STs, PGK2 for spermatozoa, and sperm quality, were performed in sections of testes of the experimental mice groups (Figure 1B). The results showed that in the testes of BU-treated mice, the percentages of positive tubules for MVH, GFRA1, SYCP3, and ACRV1 remarkably decreased or, with regard to PGK2, nearly disappeared, in comparison to CTRL. Diversely, the percentages of seminiferous tubules positive for these markers significantly increased in mice treated with BU+Npre, such that the ratio of MVH-and SYCP3-positive tubules became comparable to CTRL (Figure 1B and Figure S3). Western blot (WB) analyses confirmed these patterns (Figure 1C).

**Figure 2:**
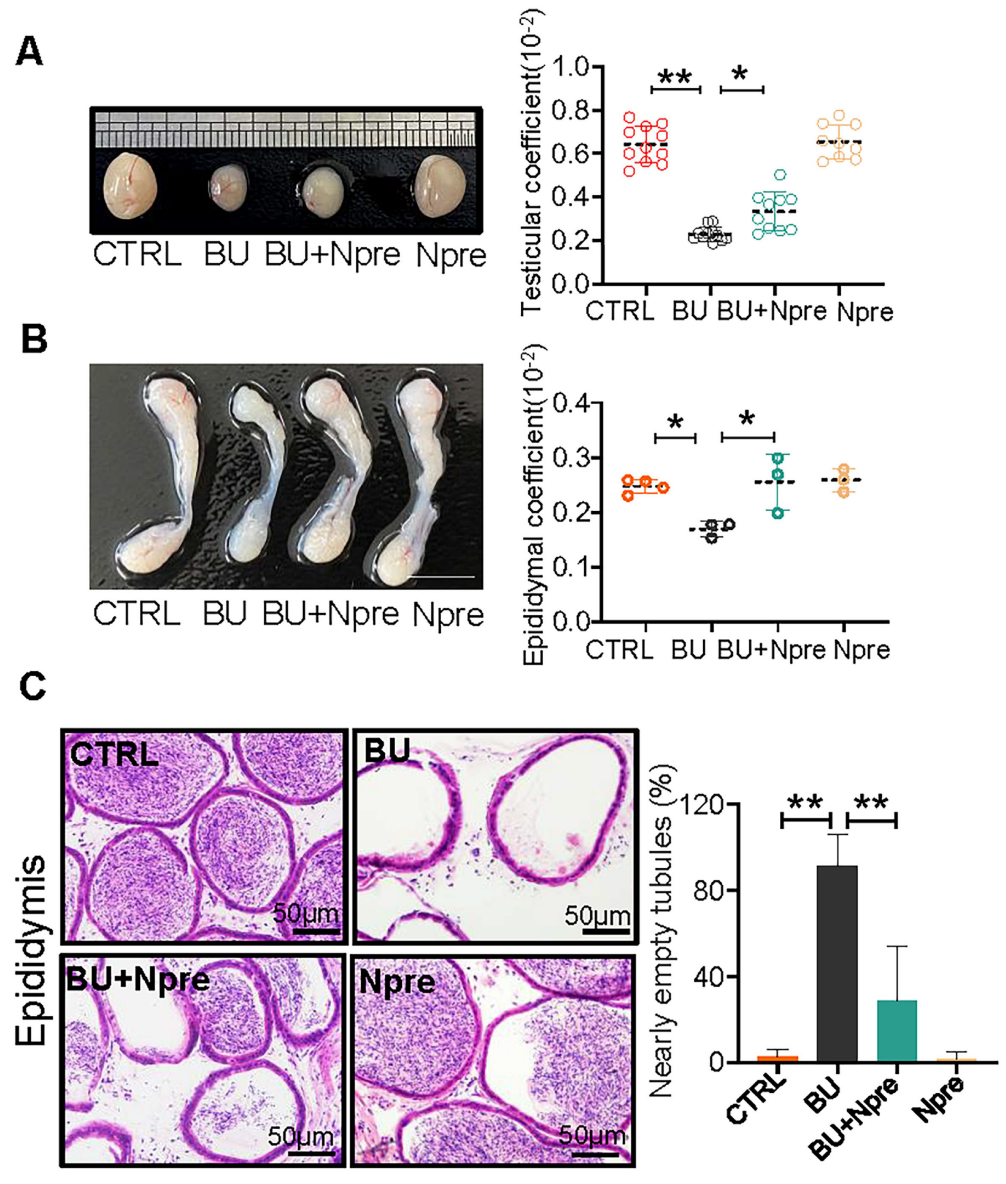
** Npre partly improves testis and epididymis size and reinstates sperm in epididymis of BU-treated mice.** (A) Representative testis size of mice of the indicated experimental groups (left). Scale bar, 1mm. And the testicular coefficient (testis/body weight) on the right. (B) Representative images of epididymis (left) and the epididymis coefficient (right) of mice. Scale bar = 5 mm. (C) Epididymis sections and proportion of nearly empty tubules of the same mice groups in A. About 10 and 4 male mice were scored for testicular and epididymis coefficient, respectively, in each group. And about 4 biological repeats in each group were used for epididymis section statistics. Data are shown as mean ± SD (^*^P < 0.05; ^**^P < 0.01).

Moreover, Npre supplementation partially restored the size and coefficient of testis and epididymis (Figure 2A-B), and reinstated sperm presence in the epididymis of BU-treated mice (Figure 2C). Encouragingly, Npre supplementation also attenuated the damage to the blood-testis barrier (BTB) and reduced the level of pro-inflammatory cytokines in the epididymis induced by BU (Figure S4 and Figure S5A).

### Npre improves semen parameters and partially restores the sperm fertilization capability of busulfan-treated mice

**Figure 3:**
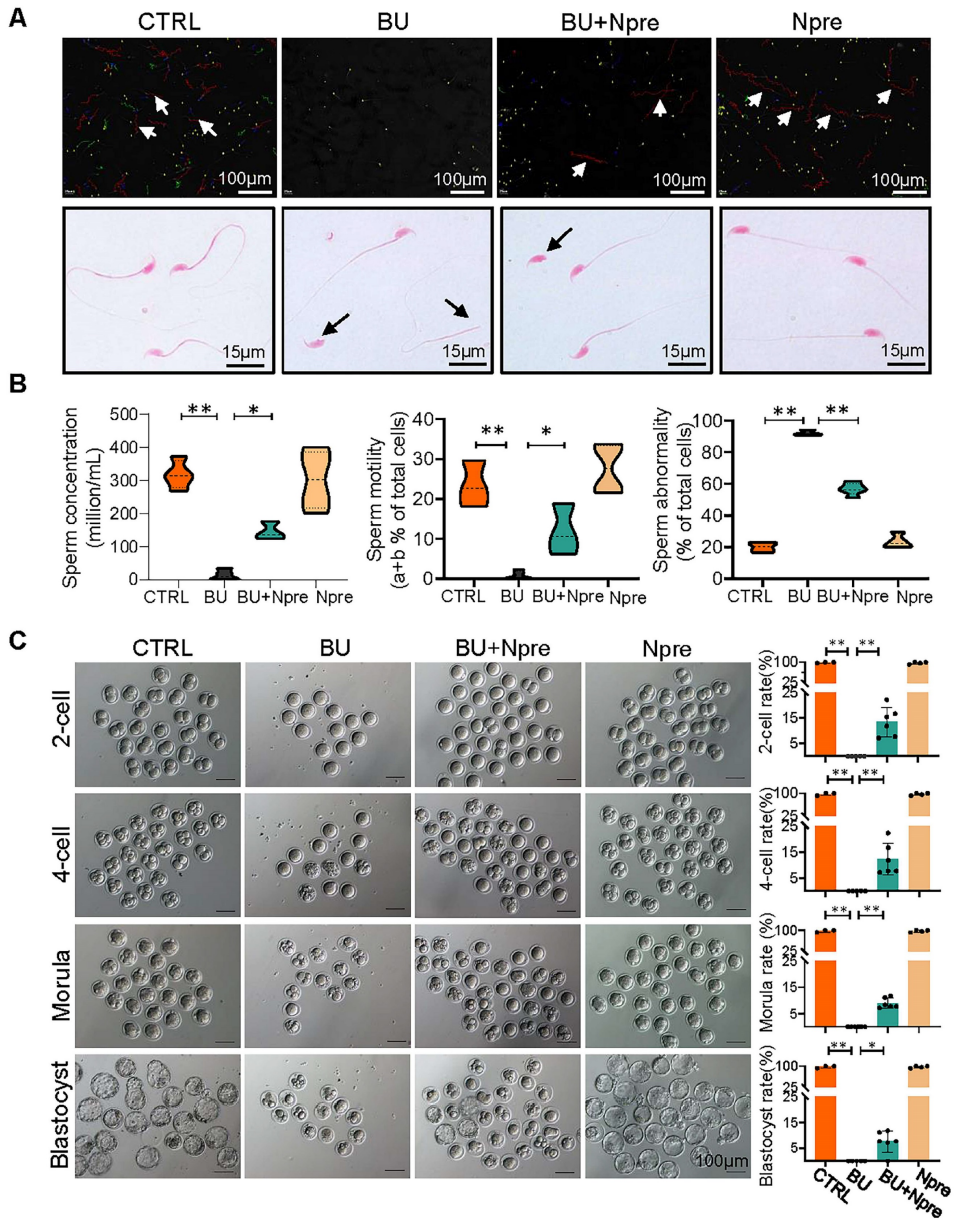
**Npre improves semen parameters and sperm fertilization capability of BU-treated mice.** (A) Representative images of sperm of the indicated male groups in the CASA system. In the upper picture, the red lines pointed by the arrows are sperm that move rapidly forward (also called class a sperm), green lines represent sperm that move slowly forward (class b sperm), blue lines represent sperm that do not move forward (class c sperm), and yellow represents sperm that are extremely slow or immobile (class d sperm). And the sperm was stained with eosin (1%) to assess sperm morphology (bottom). (B) Semen analyses of the groups indicated in A. The total proportions of class a and class b sperm were calculated as the index of sperm motility. At least 5 male mice were scored in each group for semen parameters. (C)About 138 1-cell embryos were obtained from 12 females mated with CTRL and Npre males, these almost 100% (136/138) formed blastocysts, 6 females mated with BU males did not form blastocysts, whereas there are 95 1-cell embryos from 7 females mated with BU+Npre males, and about 10% 1-cell embryos (n=9/95) formed blastocysts. Scale bar=100 μm. About 3-5 male mice were used to mate with female mice in each group. Data are shown as mean ± SD (^*^P < 0.05; ^**^P < 0.01).

Next, we evaluated the effect of Npre on semen parameters and sperm fertilization capability. As shown in Figure 3A-B and Figure S5B-C, supplementation with Npre to BU-treated mice markedly rescued sperm concentration (CTRL = 316.8 ± 43.27, BU= 12.93 ± 16.37, BU+Npre = 145.2 ± 27.48, Npre = 302.1 ± 88.40), motility (CTRL =23.43 ± 5.80%, B = 0.76 ± 1.10%, BU+Npre = 11.90 ± 6.39%, Npre = 27.65 ± 6.19%), morphological abnormalities (CTRL =19.95 ± 3.89%, BU = 92.19 ± 1.76%, BU+Npre = 56.82 ± 3.89%, Npre = 23.51 ± 4.50%) and DNA fragmentation index (DFI; CTRL =4.36 ± 2.25%, BU = 29.95 ± 2.42%, BU+Npre = 17.58 ± 1.49%, Npre = 4.32 ± 1.98%).

As expected, the tubal ampullae of females mated with CTRL and Npre males contained 138 1-cell embryos from 12 plugged female, while no fertilized eggs were present in the ampullae of females mated with BU males (0/70, MII oocyte from six females). However, some 1-cell embryos were present in the ampulla of plugged females mated with BU+Npre males. Upon *in vitro* culture, 1-cell embryos from BU+Npre showed some capability to develop into blastocysts, albeit with efficiency significantly lower than that of CTRL (CTRL = 97.62 ± 4.12%, BU+Npre = 7.567 ±4.19%, Npre = 97.18 ± 3.66%) (Figure 3C). Additionally, fertility statistics, including the number of pregnant mice and living pups per litter in each group, were presented in Table S1.

### Npre resets the transcriptome of the busulfan treated mouse testes

**Figure 4:**
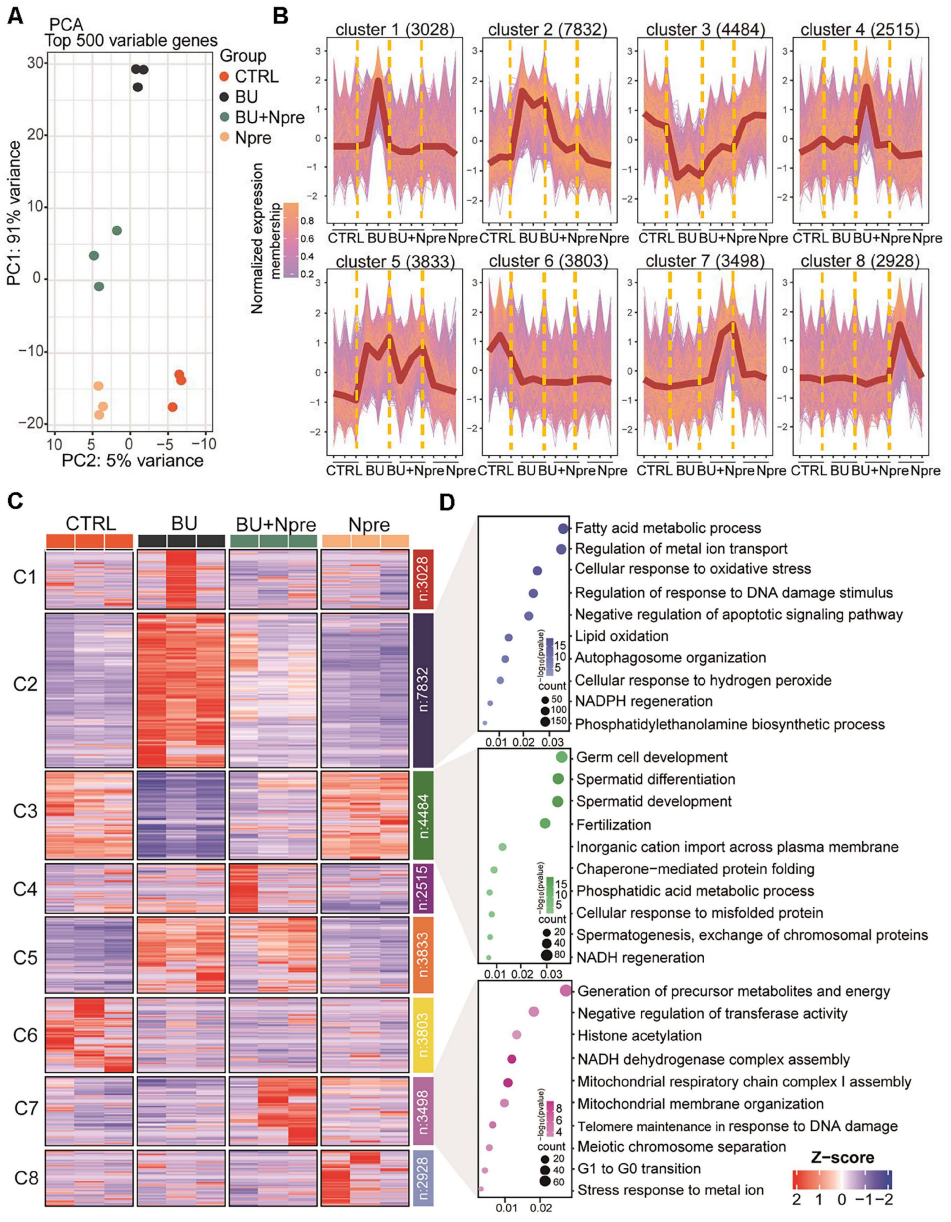
** Effect of BU and Npre on testis transcriptome.** (A) Principal component analysis (PCA) of top 500 variable genes in the testes of the indicated male groups. (B) Normalized membership representation of gene expression in the testes of the indicated groups in eight clusters. (C) Heatmap expression levels of genes of the eight clusters showed in B. (D) GO term enrichment analysis of genes of the clusters 2, 3 and 7 showed in B.

Principal component analysis (PCA) of RNA-seq data demonstrated that the BU treatment markedly altered the testis transcriptome landscape, while Npre supplementation resettled the transcriptional pattern closer to CTRL (Figure 4A).

According to the expression trends, the data were subdivided into eight clusters. Cluster data, represented as a normalized membership graph (Figure 4B) and heatmap (Figure 4C), showed that mRNA in cluster 2 and 5 were highly (cluster 2) or moderately (cluster 5) upregulated in BU-treated testes. Conversely, the drug caused downregulation of the most part of cluster 3 and 6 mRNA. Noteworthy, with the exception of cluster 6 and partly of class 5 mRNA, following Npre supplementation, deregulated expressions were resettled close to CTRL values. Intriguingly, the most part of cluster 7 mRNA appeared upregulated only in the presence of Npre, suggesting NAD^+^ stimulation of the coding genes independently of BU.

Gene Ontology (GO) analysis (Figure 4D) showed that genes coding of cluster 2 top mRNA were enriched in terms of processes highly related to ferroptosis such as “Fatty acid metabolic”, “Regulation of metal ion transport”, “Cellular response to oxidation stress”, and “Regulation of response to DNA damage stimulus”, while those of cluster 3 were enriched in spermatogenesis processes. Cluster 7 top mRNA were enriched in processes related to the “Generation of precursor metabolites and energy”, “Negative regulation of transferase activity”, “Histone acetylation” and “NADH dehydrogenase complex assembly”.

**Figure 5:**
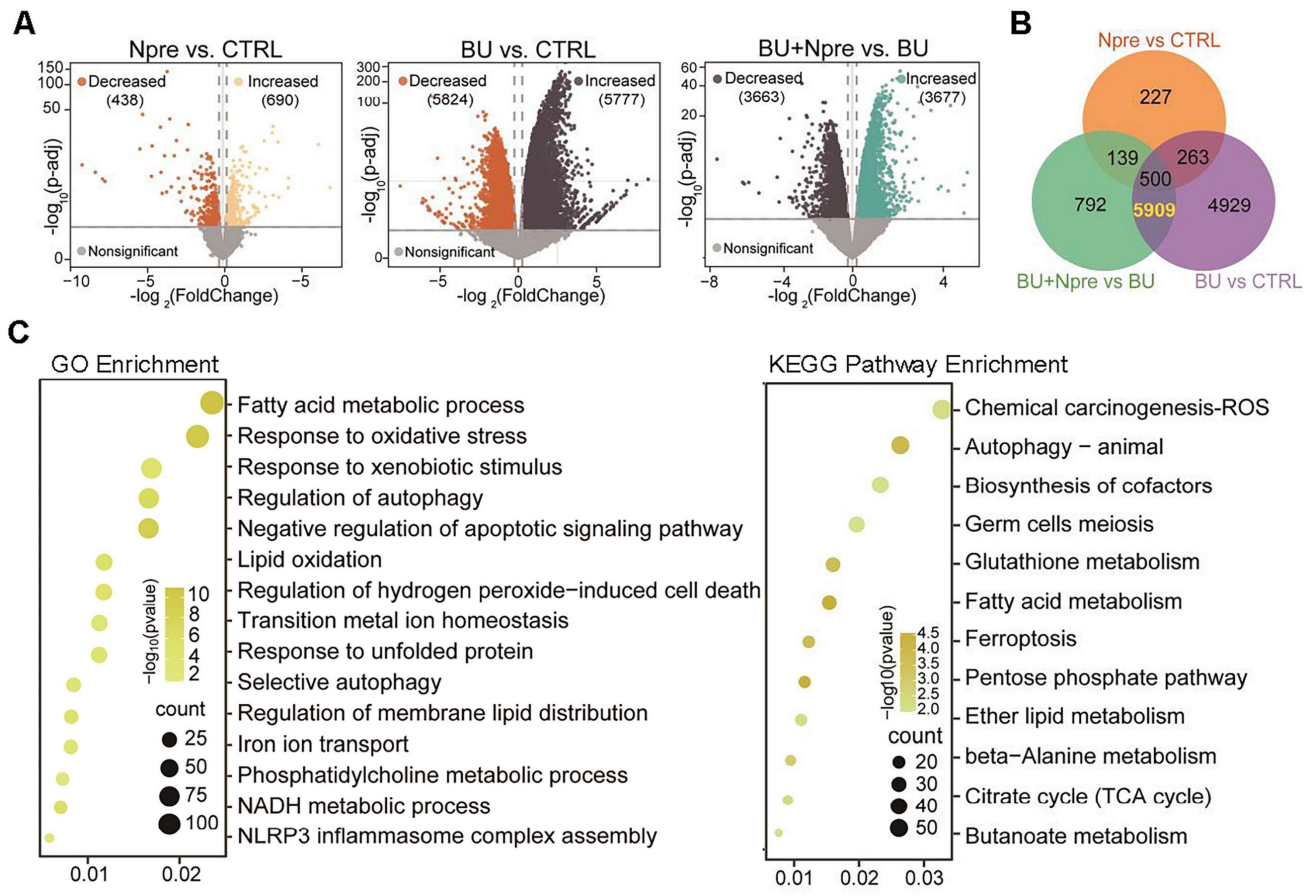
** Seq-RNA suggests ongoing ferroptosis in testes of BU-treated mice.** Comparison of volcano plots (A), Venn diagram (B) and GO and KEGG term/pathway enrichment (C) of differential expressed genes (DEGs) in CTRL, BU, BU+Npre and Npre testes.

Comparing differentially expressed genes (DEGs) in the testes of the experimental groups, the Volcano plot showed 5777 upregulated and 5824 downregulated genes in BU *vs* CTRL, 690 upregulated and 438 downregulated genes in Npre *vs* CTRL and 3677 upregulated and 3663 downregulated genes in BU+Npre *vs* BU testes (Figure 5A). The Venn diagram displayed that excluding 500 DEGs between Npre and CTRL, 5909 DEGs were in the intersection between BU *vs* CTRL and BU+Npre *vs* BU (Figure 5B and Table S2). GO and Kyoto Encyclopedia of Genes and Genomes (KEGG) enrichment analysis of the DEGs revealed that when Npre was supplementation to BU-treated mice, the ferroptosis pathways including oxidative stress, autophagy, lipid oxidation, iron transport, and glutathione metabolism were among the processes most affected in the testis (Figure 5C). Figure S6A shows box plots of representative genes related to oxidative stress (*Gpx4*, *COX2* and* Slc3a2*), lipid oxidation (*Acsl4*, *Acsl3*, *Alox12* and *Alxo15*), and iron transport (*Fth1*, *Tfrc* and* Ncoa4*) resettled to CTRL expression levels by Npre supplementation.

### Npre alleviates spermatogenic cell ferroptosis in busulfan-treated mice

**Figure 6:**
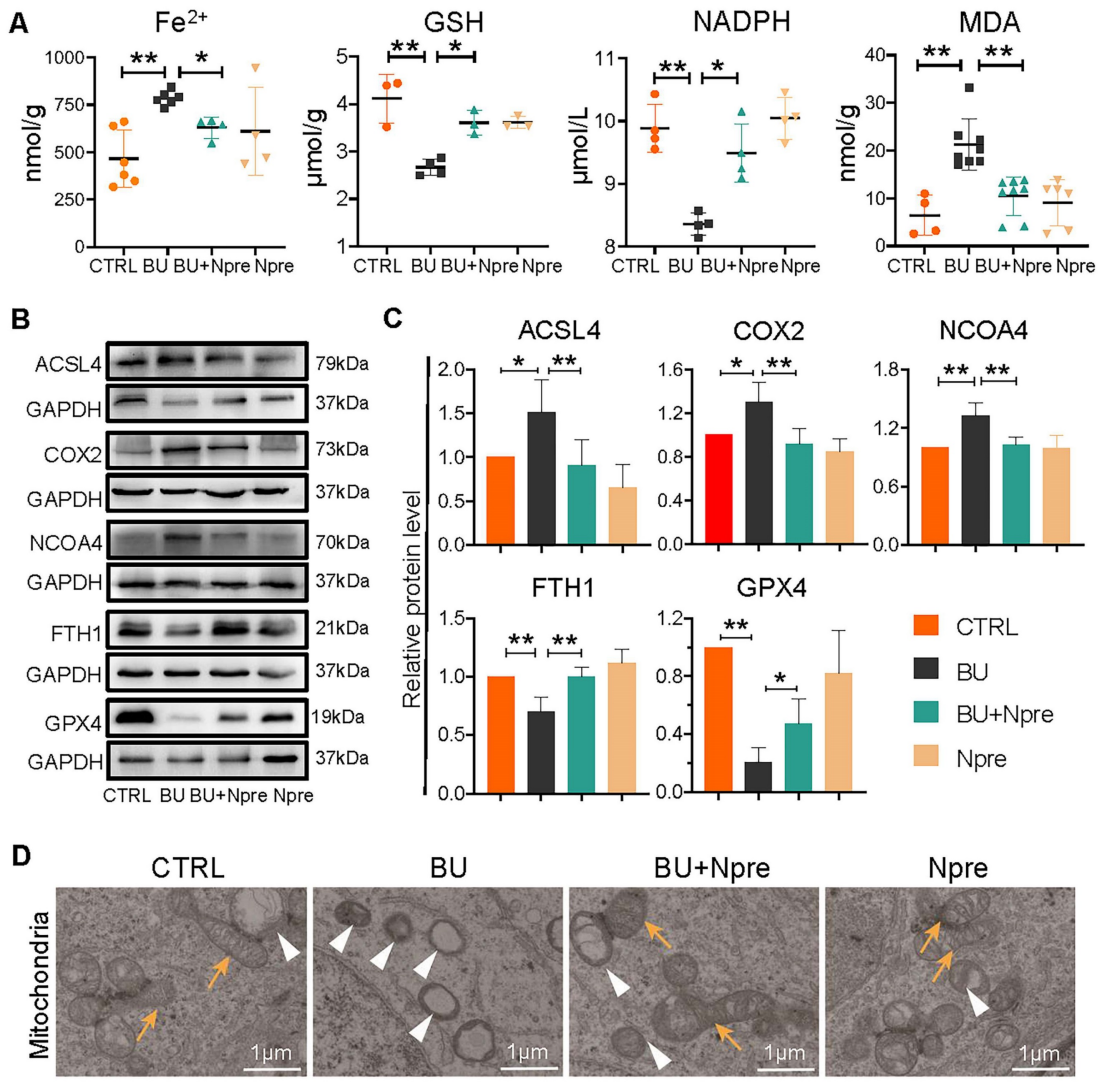
** Effect of BU and Npre on testis ferroptosis.** (A) Concentrations of ferroptosis biomarkers in testes from the indicated groups. (B) Representative protein bands of ferroptosis biomarkers in testes from the indicated groups and their quantification relative to GAPDH. Each biological repeat came from at least three to five male mice. (D) TEM images of mitochondrial morphologies in each group. Note, in CTRL and Npre group, most of the mitochondria are intact ellipse with many inner cristae, in BU group, mitochondria become shrunken and were almost devoid of cristae. Arrows, normal mitochondria; arrowheads, abnormal mitochondria. Scale bar = 1 μm. The results include three independent replicates and are showed as mean ± SD (*P < 0.05; **P < 0.01).

To support the indication coming from RNA-seq that BU increases ferroptosis in spermatogenesis and Npre are able to reduce such process, we measured the testis levels of ferroptosis biomarkers such Fe^2+^, glutathione (GSH), nicotinamide adenine dinucleotide phosphate (NADPH), and malondialdehyde (MDA). The results revealed significantly higher concentrations of Fe^2+^ and MDA, and lower GSH and NADPH in BU than in CTRL testes and confirmed that Npre supplementation to BU-treated mice resettled the levels of these compounds close to those of CTRL (Figure 6A). In line with these, WB analyses showed increased quantity of Acyl-CoA synthetase long-chain family member 4 (ACSL4), nuclear receptor coactivator 4 (NCOA4), and prostaglandin endoperoxide synthase 2 (COX2), which are biomarkers of ongoing ferroptosis, and decreased amount of Ferritin Heavy Chain 1 (FTH1) and GPX4, negative regulators of ferroptosis, in BU-testes and reversion to CTRL levels following Npre supplementation (Figure 6B-C). Using transmission electron microscopy (TEM), we observed that the mitochondrial morphology was atrophied, the outer membrane was dense, and mitochondrial cristae was almost devoid after BU treatment (Figure 6D). Npre supplementation partially restored the abnormal mitochondrial morphology caused by BU. These findings indicate that Npre treatment could alleviate ferroptosis caused by BU.

The crucial involvement of ferroptosis in the deleterious effect of BU on spermatogenesis was further supported by the effects of supplementation with 1 mg/kg ferrostatin-1 (Fer-1), an inhibitor of ferroptosis, to BU mice. Actually, in these mice, Fer-1, as Npre supplementation, resettled testicular coefficient, sperm concentration and motility, and MDA level close to CTRL values (Figure S6B-C). Similarly, following Fer-1 supplementation, in the BU testes, the levels of MVH and SYCP3 proteins, as well as those of GPX4 and ACSL4, were recovered to CTRL values (Figure S6D).

### The effects of Npre on ferroptosis and spermatogenesis partly depend on SIRT2-PGC-1α/ACLY pathway

Since Npre supplementation results an increase in blood NAD^+^ level (Figure S1B), and the GO terms of cluster 7 suggested upregulation of NAD^+^ pathways in both Npre and BU+Npre (Figure 4D), and considering that sirtuins, a family of NAD^+^-dependent deacetylases, play a crucial role in ferroptosis and spermatogenesis 29, 30, we investigated whether these latter were involved in the beneficial effects exerted by Npre on spermatogenesis.

**Figure 7:**
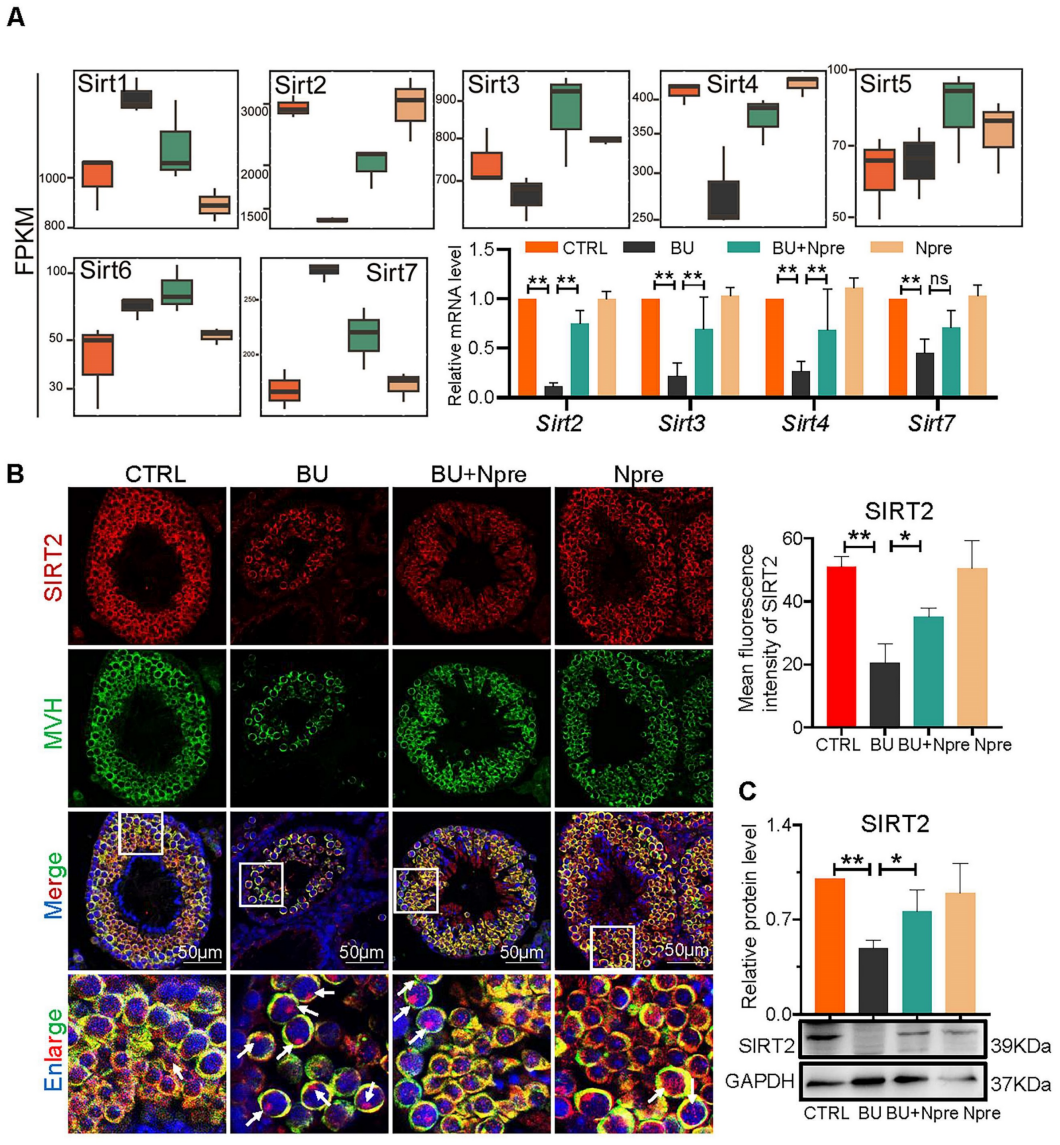
** Involvement of sirtuins in the beneficial effect of Npre supplementation on spermatogenesis of BU-treated mice.** (A) Box plots of FPKM evaluation of *Sirt1*-*7* expression and RT-qPCR of mRNA amount of *Sirt2*, *Sirt3*, *Sirt4* and *Sirt7* in testes of the indicated groups. (B) Left, representative IF for MVH and SIRT2 in sections of seminiferous tubules of testes of the indicated groups (white arrow, SIRT2 located in the nucleus, nuclei counterstained with Hoechst 33342); Right, mean fluorescence intensity of the SIRT2 cytoplasm staining measured in single germ cells. (C) Representative WB and quantitative evaluation of SIRT2 expression relative to GAPDH in testes of the indicated groups. Each result of testicular section was derived from at least 3 to 6 mice. The results include three independent replicates and are showed as mean ± SD (^*^P < 0.05; ^**^P < 0.01; ns=not significant).

Analyzing RNA-seq, Fragments Per Kilobase Million (FPKM) box plot graphs showed that *Sirt1*-*7* were all expressed in mouse testis, and major changes in *Sirt2*, *Sirt3*, *Sirt4* (decreased expression) and *Sirt7* (increased expression) in testes of BU mice were resettled to levels comparable to CTRL by Npre supplementation (Figure 7A). RT-qPCR and WB analyses confirmed such results for *Sirt2*, *Sirt3*, *Sirt4* and* Sirt7* (Figure 7A, C and Figure S7). Likewise, IF staining showed that SIRT2 staining, mainly localized in the cytoplasm of germ cells, declined in BU testis but reverted to CTRL levels following Npre supplementation (Figure 7B). Given the above results, and its important role in lipid metabolism and oxidative stress, SIRT2 was further investigated in the following experiment.

**Figure 8:**
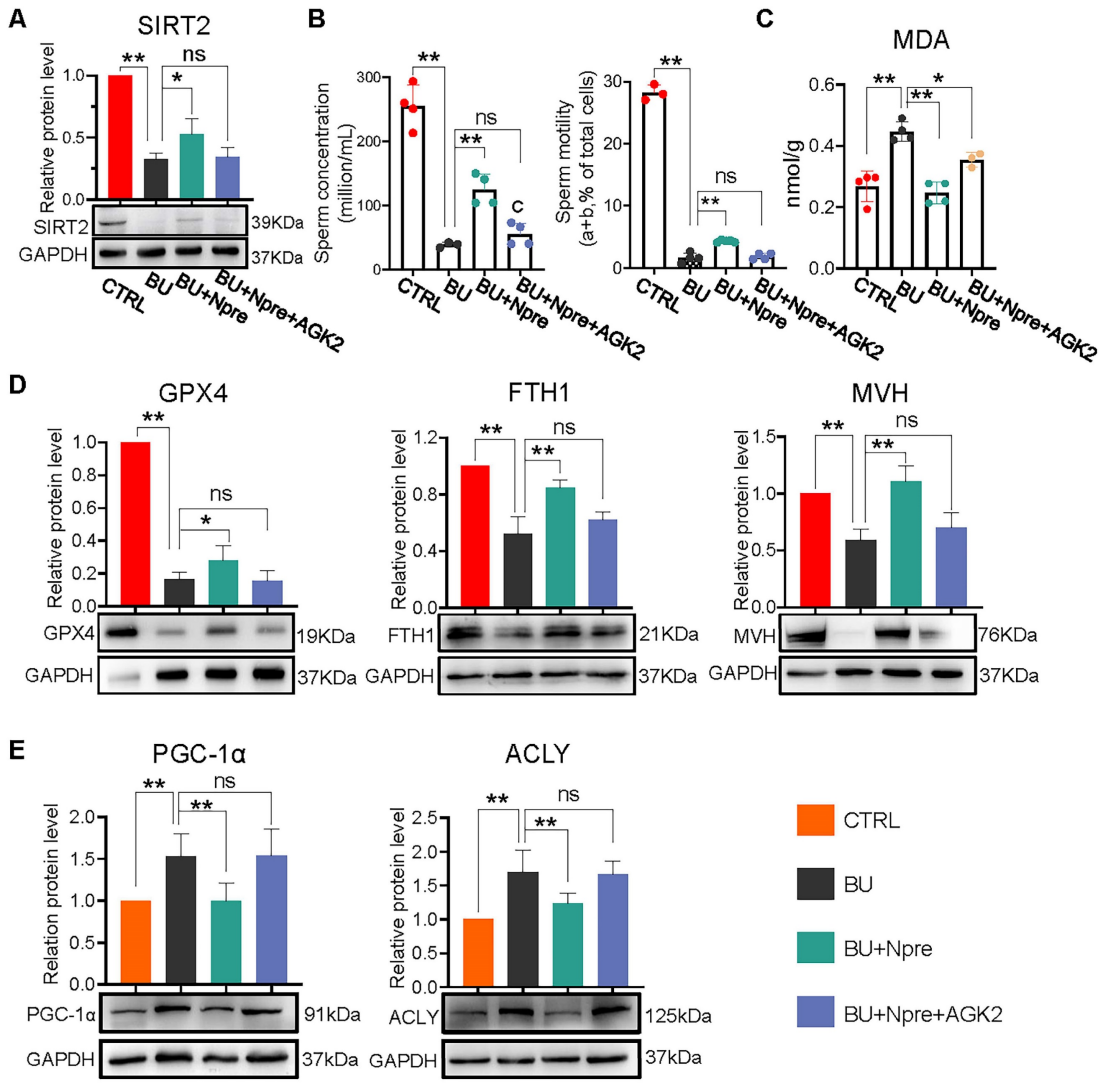
** Inhibition of SIRT2 lessens the beneficial effect of Npre supplementation on ferroptosis and spermatogenesis of BU-treated mice.** AGK2 injection (SIRT2 inhibitor) reduces the SIRT2 protein level (A) and counteracts the effect of Npre on number and motility (B) of sperm of BU-treated males (a+b are sperm moving forward). (C) AGK2 resets the level of the ferroptosis marker MDA in the BU+Npre testes nearly to that found in BU-treated mice. (D) Representative WB and quantification levels of GPX4, FTH1 and MVH, relative to GAPDH, in the testes of the indicated groups. (E) Representative protein bands and their quantitative expression of PGC-1α and ACLY in testes from the indicated groups relative to GAPDH. Testis samples were taken from at least 3 to 6 mice in each group, and the results include three independent replicates and are showed as mean ± SD (*P < 0.05; **P < 0.01; ns=not significant).

We next used AGK2, an inhibitor of SIRT2, to further investigate whether the beneficial effects of Npre on spermatogenesis in BU mice depended on this deacetylase. As a matter of fact, when AGK2 was injected in BU+Npre mice, the recovery effect of Npre on SIRT2 level in testis, sperm concentration and motility, which were depleted by BU, was almost abolished (Figure 8A-B). Moreover, anti-ferroptosis effects such as reduced MDA and increased GPX4 and FTH1 levels in the testis, and beneficial consequence on spermatogenesis testified by MVH and SYCP3 expression exerted by Npre were mitigated by AGK2 (Figure 8C-D and Figure S8A-B). To clarify the mechanism of SIRT2 regulation of ferroptosis, we detected the expression levels of key proteins involved ferroptosis, including peroxisome proliferators-activated receptor γ coactivator l-alpha (PGC-1α, a transcriptional coactivator regulated mitochondrial biogenesis and fatty acid oxidation), ATP-citrate lyase (ACLY, a lipogenic enzyme), 6-phosphogluconate dehydrogenase (G6PD, a key enzyme in the pentose phosphate pathway ) and nuclear factor erythroid 2-related factor 2 (NRF2, a transcription factor for oxidative stress), and the results showed that decreasing the expression of SIRT2 could offset the positive effects of Npre on PGC-1α and ACLY, but it did not affect the up-regulation induced by Npre on NRF2. In addition, BU treatment did not yield a significantly change in the protein level of G6PD (Figure 8E and Figure S8C). These findings suggested that Npre potentially modulates ferroptosis level in the testis through the regulation of the SIRT2-PGC-1α/ACLY pathway.

## Discussion

As mentioned in Introduction, NAD^+^ is a coenzyme involved in the regulation of multiple cells signaling often related to metabolic and energy pathways. Decreased levels of systemic and intracellular NAD^+^ have been associated with numerous diseases, which the administration of NAD^+^ precursors, under certain conditions, has shown the potential to ameliorate or alleviate 31.

In our study, a combination of NA and NAM supplementation (Npre) was employed with the aim to counteract male infertility caused by a single injection of BU. Such condition, occurring as a consequence of DNA alkylation induced by the drug, and the competence of 1-cell embryos obtained from oocytes fertilized by sperm from BU+Npre males to develop into blastocysts remained, however, largely lower than CTRL. Thus, indicating that damages caused on germ cells by BU were not completely recovered.

Although growth factors such as SP, LGF and G-CSF have demonstrated a propensity to promote the spermatogenesis recovery by reactivating the spermatogonia compartment 9-11, other compounds including retinoic acid, melatonin, and alginate oligosaccharides have shown efficacy in improving spermatogenesis in BU-treated mice through reduction of testis ROS production or meliorating general metabolism 5, 12, 32. In this regard, mounting evidences indicate that oxidative stress primarily induces cell death through ferroptosis. This emerging form of cell demise has been increasingly implicated in a range of pathological processes, including Parkinson's disease, liver fibrosis, and reproductive disorder 33-35. As a matter of fact, ferroptosis is caused by intracellular iron overload, ROS accumulation, and lipid peroxidation. Cells with ferroptosis are characterized by rupture of cytomembrane and mitochondrial outer membrane, reduction in mitochondria volume and cristae 36-38. Moreover, abnormal protein levels of GPX4, COX2, ACSL4, NCOA4, FTH1, as well as alterations in the content of GSH and NADPH, serve as biomarkers of ferroptosis. Presently, a connection between ferroptosis and male reproduction disorder has been established 39. In fact, some studies have reported that the induction of testicular oxidative stress leading to ferroptosis by substances such as arsenite, copper, and PM2.5 particles 33, 40, 41, while Di (2-ethylhexyl) phthalate has been shown to induce ferroptosis via HIF-1α/HO-1 signaling pathway and transferrin receptor 1, 42. Relevant for the present work, Zhao et al. recently reported that ferroptosis is involved in BU-induced oligospermia in mice and that such effect is mediated by inhibition of Nrf2-GPX4 (FPN1) signaling pathway 2. Here, we found that genes related to ferroptosis were mis-expressed in infertile mice treated with BU but returned to normal expression levels after Npre supplementation. At the same time, intersection DEGs were enriched to a number of cellular processes associated with ferroptosis, including “lipid oxidation”, “oxidative stress”, “GSH metabolism”, and “iron ion transport”. Moreover, WB results showed that Npre attenuated the changes in the concentration of ferroptosis markers such as Fe^2+^, GSH, NADPH, GPX4 and ACSL4 caused by BU in the testis. All together these results, in line with those reported in other recent studies, support the notion that NAD^+^ alleviates ferroptosis 43, 44.

Interestingly, adequate level of NAD^+^ is crucial to sustain the activity and concentration of sirtuin, a family of NAD^+^-dependent histone deacetylases involved in cell metabolism and capable of regulating numerous cellular functions 30, 45, 46. Among sirtuins, SIRT2 exhibits the highest expression level in the testis and can undergo translocation from the cytoplasm to the nucleus 46. Transgenic models provide strong evidence that sirtuins are involved in spermatogenesis by influencing specific functions of male germ cell, Sertoli cells and Leydig cells (for a review) 30. Besides, showing that* Sirt1*-*7* were expressed in mouse testis and that major changes in *Sirt2*, *Sirt3*, *Sirt4* (decreased expression) and *Sirt7* (increased expression) occurred in testes of BU mice, we found that Npre supplementation to BU treated mice resettled the testicular levels of sirtuins nearly to those of CTRL values. Moreover, administration of an inhibitor targeting SIRT2 substantially reversed the beneficial effect of Npre on spermatogenesis in BU-treated males. These findings suggest that Npre supplementation efficiently improves spermatogenesis, at least in part, via SIRT2.

Currently, limited studies investigating the role of SIRT2 in ferroptosis, especially within the context of the testis. According to Zhang's studies, SIRT2 exerts an inhibitory effect on ferroptosis by modulating the expression of GPX4 and ACSL4 proteins, thereby suppressing levels of iron ions and lipid peroxidation 47. In line with these, here, our study observed that the up-regulation of GPX4 and the down-regulation of ACSL4 caused by Npre were counteracted upon suppression SIRT2 expression. A growing number of researches have highlighted that the essential role of SIRT2 in lipid metabolism and oxidation, oxidative stress, and Fe^2+^ transport 29, 48, suggesting its potential involvement in regulating various biological processes associated with ferroptosis. In terms of lipid metabolism, some studies have reported that SIRT2 decreased the PGC-1α protein level, which further inhibits fatty acid oxidation 49. Also, SIRT2 reduced the level of ACLY, thereby modulating the conversion of citric acid to lipids 50. In terms of antioxidant and redox signaling, SIRT2 has been reported to activate G6PD expression and promote antioxidant ability 51, while also regulating iron ion export and redox imbalances by modulating NRF2 52. Our results showed that while BU treatment did not significantly affect the G6PD protein level, but caused the up-regulation of PGC-1α and ACLY, and reduced the protein level of NRF2. Furthermore, reducing SIRT2 expression with AGK2 could interfere with the positive effect of Npre on PGC-1α and ACLY, but not NRF2.

Furthermore, a single-cell transcriptomic analysis conducted ontesticular samples from 17 patients with non-obstructive azoospermi (NOA), and found that the ferroptosis marker *Gpx4* and *Fth1* were down-regulated, *and Acsl4* was up-regulated in most patients compared with controls 53. These results suggest that aberrations in ferroptosis marker genes are present in a subset of NOA patients. Apart from this, Huang et al. found downregulation of SIRT2 in undifferentiated spermatogonia and some spermatocytes of NOA patients compared to healthy males 54. These reports align with our results, and collectively indicate a potential association between ferroptosis and spermatogenesis disorders. However, further investigation is warranted to elucidate the mechanism.

In conclusion, our findings indicate that Npre are able to promote the restoration of spermatogenesis in BU-treated mice by modulating the SIRT2-PGC-1α/ACLY pathway, thereby protecting spermatogenic cells from ferroptosis. This highlights the potential of Npre supplementation as novel strategy to ameliorate conditions of male infertility including cancer therapy based on alkylating compounds.

## Materials and Methods

### Mice

In all experiments, three-week old ICR male mice with confirmed fertility were used, and the mice were housed at a suitable temperature (22-24°C), a light/dark cycle for 12 h, and allowed *ad libitum* access to food and water. All animal treatments were strictly complied with the Animal Care and Ethical Committee of Qingdao Agricultural University (Approval No. 2019-036).

### Treatments and mice groups

At least ten mice were randomly assigned to control (CTRL) group and various treatment groups as follow (also refer to Figure 1A). BU mice, receiving a single intraperitoneal injection of 20 mg/kg BU (B2635; Sigma, China); BU+Npre mice, receiving BU as described above and NAD^+^ precursors, NA and NAM (59-67-6 and 98-92-0; Tianjin Zhongrui Co., Ltd, Chin), mixed in equal ratio and administered by gavage from the day after BU injection for five weeks; Npre mice, receiving only NA+NAM; BU+Fer-1 mice, receiving BU as described above and intraperitoneal injection of 1 mg/kg Fer-1 (SML0583; Sigma), three times a week for five weeks from the day after BU injection; Fer-1 mice, receiving only Fer-1; AGK2 mice receiving BU as described above and daily intraperitoneal injection with 1 mg/kg *Sirt2* inhibitor AGK2 (S7577; Selleck, USA), for five weeks from the day after BU injection.

### Testicular and epididymal coefficients

Mice were weighed before sacrifice, and bilateral testes and epididymal were immediately removed and weighed. The formula for calculating testicular and epididymis coefficient was as follow: Testicular Coefficient = Bilateral testicular weight (g) / Mice weight (g) × 100%; Epididymal Coefficient = Bilateral epididymal weight (g) / Mice weight (g) × 100%.

### Evaluation of sperm quality

Cauda epididymis was dissected and minced in DMEM/F12 (SH30023.01B; Hyclone, China) at 37°C to release and disperse sperm. Sperm number and motility were assessed using a computer-assisted sperm assay (CASA) system 55. Sperm drops were placed onto a slide and stained with eosin (1%) for 2 hours to assess sperm morphology. Sperm DNA fragmentation index (DFI) was detected using the sperm chromatin dispersion (SCD) assay, (BRED-002, BRED Life Science, China) with the procedure strictly following the instructions of manufacturer.

### Histopathological analysis and immunofluorescence

The testis, epididymis, liver, intestine and ovary were fixed in 4% formaldehyde for 24 hours and paraffin embedded following standard procedures. Subsequently, the samples were trimmed, sliced (5 μm), and stained with hematoxylin and eosin (H&E) for histopathological analysis. The surfaces of over 80 typical seminiferous tubules were calculated using image J software.

For IF analysis, testes were fixed, paraffin embedded and sliced as described above. Sections were then dewaxed, rehydrated and subjected to antigen retrieval. Briefly, samples were blocked with BDT (containing 10% goat serum diluted and 3% BSA) for 40 min at r.t. and incubated overnight with primary antibody (Table S3). The next day, after TBST washing, secondary antibody (Table S3) incubation at 37°C for 50 min was carried out and nuclei stained with Hoechst33342 (C1022; Beyotime, China) for 5 min at 37°C. Finally, sections were rinsed in PBS for three times and sealed with an anti-fluorescence attenuation quenching agent (I033; Boster, China). Slides were observed and photographed using a fluorescence microscope (BX51; Olympus, Japan). Image J software was used to measure the mean fluorescence intensity per cell.

### ELISA

Serum concentration of hormones including INH-B (HS106; Jinma, China), T (JM-02852M2; Jingmei Biological, China), FSH (JM-02838M2; Jingmei Biological), IL-6 (JM-02446M2, Jingmei Biological), IL-10 (JM-02459M2, Jingmei Biological) and NAD^+^ (JB156; Jinma), were evaluated using the ELISA kit mentioned above. As well as IL-6 and IL-10 levels in the epididymis were measured using the same ELISA kits. NADPH concentration in testes was evaluated using S155 ELISA kit from Jinma (China). Procedures have been previously described in detail 56, 57.

### *In vitro* embryo culture

Natural matings between mature females (over 6-7 week of age) and males were used to supply the 1-cell embryos. About 30 1-cell embryos were collected from the ampulla of the fallopian tube and cultured in 70 μL KSOM medium (M1430; Aibei, China) at 37 °C with 5% CO_2_ in air with maximal humidity. The number of 2-cells, 4-cells, morulae and blastocysts were scored throughout the culture up to 96 hours of incubation.

### Detection of Fe^2+^, GSH and MDA contents in testes

Fe^2+^, GSH and MDA concentrations in testes were detected using ADS-W-QT027, ADS-W-G001, ADS-W-YH002 kits from Jiangsu Aidisheng Biological Technology Co., Ltd (China), respectively. Briefly, 500 μL of extraction solution was added to 0.05g of testis tissues. After grinding and centrifugation, the supernatant was collected and mixed with kit reagents for 15 min (Fe^2+^ assay), 5 min (GSH assay) and 30 min (MDA assay) and absorbance at 562 nm (Fe^2+^), 412 (GSH), 532 nm and 600 nm (MDA) measured by multi-function microchannel plate detector (Cytation; Biotech, USA).

### RNA-Seq and data analysis

Testes were sent to Novogene Co. Ltd ((Berry, Beijing, China) for RNA‐Seq library preparation and sequencing. The sequencing data were quality-controlled and filtered by FastQC (v0.11.9) and FASTP (v0.22.0) software, and the gene expression levels in each group calculated with FeatureCounts (v1.6.3) software. Genes were grouped according to the expression trend using ClusterGVis (R package, v0.04). Finally, the DEGs among groups were screened and the ClusterProfiler (R package, v4.6.0) used for GO analysis and KEGG analysis.

### Transmission Electron Microscopy (TEM)

The fresh testicular tissues were obtained and fixed at 4°C for 24 hours with 2.5% glutaraldehyde. Following to the standard procedure of TEM, the sample was coated with resin. The ultrathin sections were stained with lead citrate and uranyl acetate, and the images were captured by HT7700 transmission electron microscopy (Hitachi, Tokyo, Japan).

### Western blotting

Testes were vortexed in RIPA lysis buffer (Beyotime, P0013C) on ice for 2 min. Sodium dodecyl sulfate (SDS) was then added to the extracts, and the samples were boiled for 5 min. Western blotting was carried out following standard procedures. Briefly, proteins were electrophored in 12% SDS-PAGE gel at 100 V constant voltage and transferred to PVDF membrane (ISEQ00010; Millipore, USA). The PVDF membranes were blocked with 5% BSA diluted by TBST for 2 hours at room temperature and incubated with primary antibody (Table S3) at 4°C overnight and HRP-conjugated secondary antibodies (Table S3) for 1 hours at room temperature. The signal was detected using an ECL Plus (180-506; Tanon, China), and analyzed using AlphaView SA software (ProteinSimple, USA); GAPDH serves as reference protein.

### RNA extraction and quantitative real time PCR (RT-qPCR)

SPARKeasy Improved Tissue/cell RNA kit (AC0202; Sparkjade, China) was used to extract total RNA from testes. Equal amounts of RNA (300μg/mL) were reverse transcribed to cDNA using the SPARK script II RT Plus Kit (AG0401; Sparkjade) and RT-qPCR was carried out on a CFX96 Real-Time System instrument, including SYBR Premix Ex Taq™ II (Q711-02; Vazyme, China), RNase-free-H_2_O and primers (Table S4).

### Statistical analysis

All experiments and statistical data include at least three biological replicates, expressed as the mean ± standard deviation (SD). Data were analyzed using analysis of variance ANOVA. ^*^P < 0.05 = significant difference, and ^**^P < 0.01 = highly significant difference.

## Supplementary Material

Supplementary figures and table 1.

Supplementary table 2.

Supplementary table 3.

Supplementary table 4.

## Abbreviations

ACSL4: Acyl-CoA synthetase long-chain family member 4
ACLY: ATP-citrate lyase
BU: busulfan
BTB: blood-testis barrier
COX2: prostaglandin endoperoxide synthase 2
DEGs: differentially expressed genes
DFI: DNA fragmentation index
FSH: follicle-stimulating hormone
Fer-1: ferrostatin-1
FTH1: Ferritin Heavy Chain 1
G6PD: 6-phosphogluconate dehydrogenase
GSH: glutathione
GPX4: glutathione peroxidase 4
INH-B: inhibin B
IF: Immunofluorescence
MDA: malondialdehyde
NAD^+^: nicotinamide adenine dinucleotide
Npre: nicotinamide adenine dinucleotide (NAD^+^) precursors
NADPH: nicotinamide adenine dinucleotide phosphate
NA: Nicotinic acid
NAM: Niacinamide
NMN: Nicotinamide mononucleotide
NR: Nicotinamide riboside
NCOA4: nuclear receptor coactivator 4
NRF2: nuclear factor erythroid 2-related factor 2
PCA: principal component analysis
PGC-1α: peroxisome proliferators-activated receptor γ coactivator l-alpha
RT-qPCR: real-time quantitative PCR
ROS: reactive oxygen species
SSCs: spermatogonia stem cells
SPGs: spermatogonia
SPCs: spermatocyte
STs: haploid spermatids
SIRT2: sirtuin 2
T: testosterone
WB: western blotting
