# Supplementary material for: NAD+ precursors promote the restoration of spermatogenesis in busulfan-treated mice through inhibiting Sirt2-regulated ferroptosis
Source: Theranostics. 2024 Apr 15;14(6):2622–36. doi: 10.7150/thno.92416 (PMC11024856; doi:10.7150/thno.92416)
Supplement: Supplementary file 1 — Supplementary figures and table 1. [file thnov14p2622s1.pdf]

Supplementary Figures

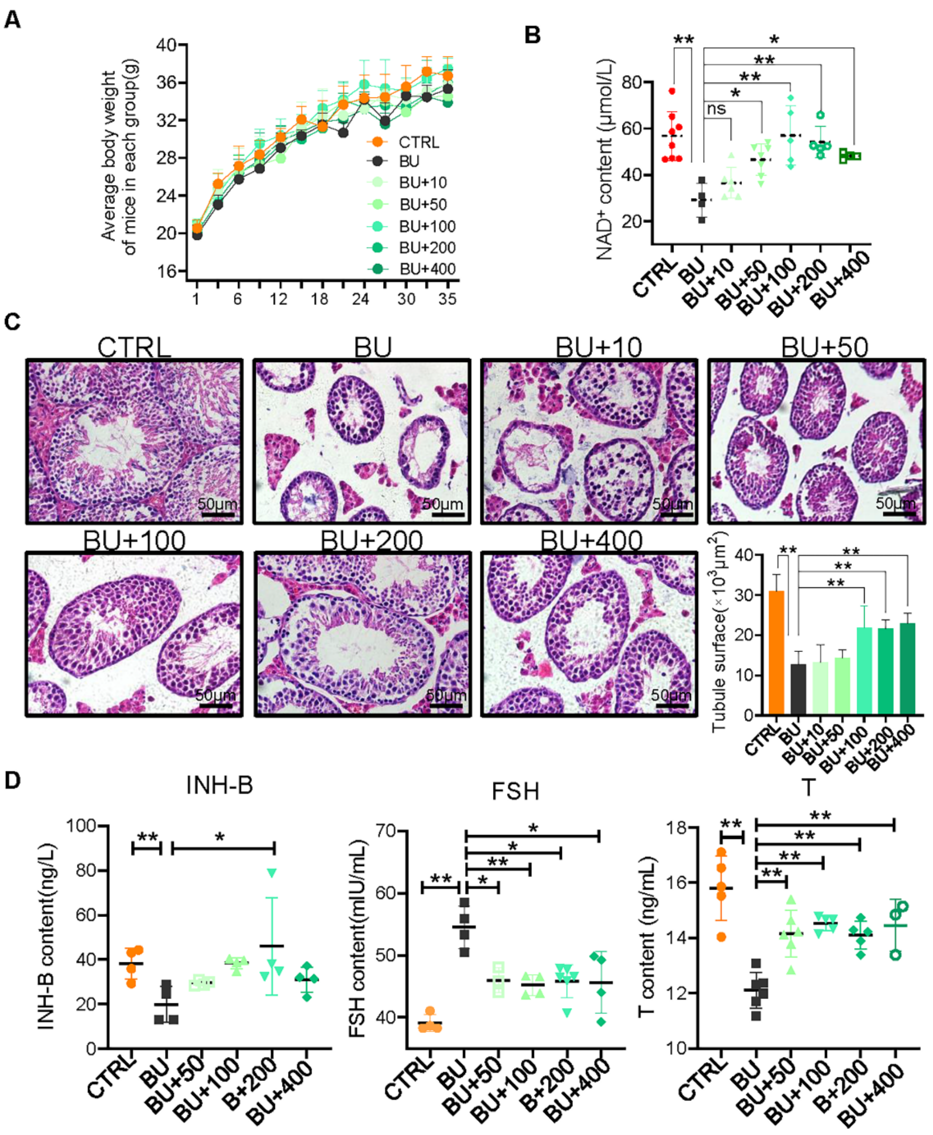

Figure S1. Optimum concentration selection for Npre treatment

Effects of single 20 mg/kg injection of BU and ameliorative impact of 10, 50, 100, 200, 400 mg/kg Npre gavage supplementation on body weight (A), blood NAD^+ concentration (B), seminiferous tubule size/surface (in each group, at least 40 tubules of four testes from 4 males examined) (C), and blood INH-B, FSH and T concentration (D). Data from at least three independent calculations are showed as mean ± SD ($^*P < 0.05$; $^{**}P < 0.01$; ns=not significant).

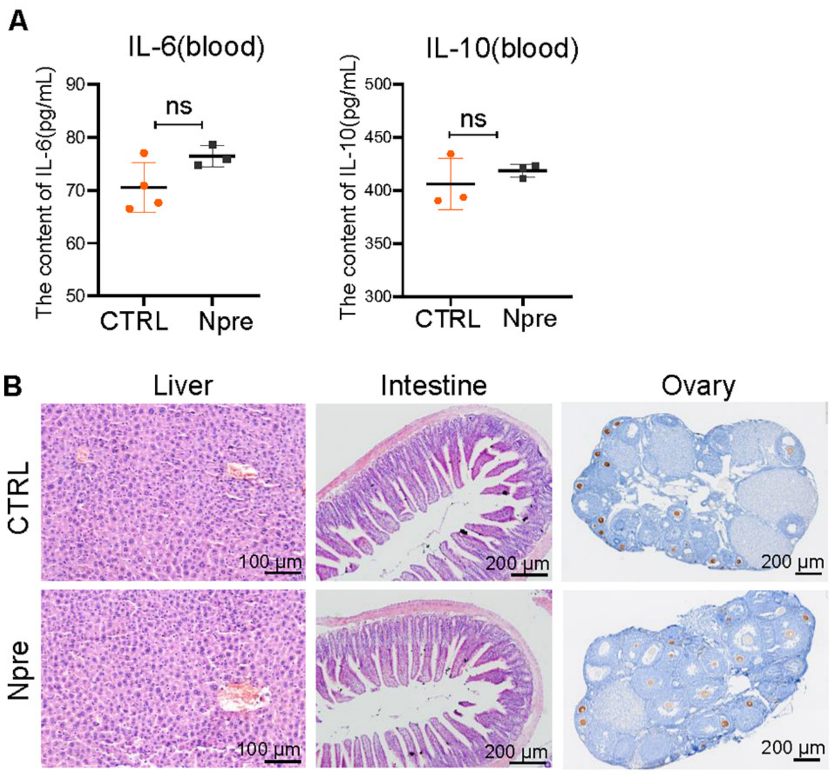

Figure S2. Effects of Npre administration on body and other organs for 35 days

(A) The content of pro-inflammatory cytokines in the blood of the indicated groups. (B) The HE and IHC images for liver, intestine and ovary. Scale bar = 100 μm, 200 μm and 200 μm, respectively. The blood of 3 male mice were collected for measuring the content of pro-inflammatory cytokines in CTRL- and Npre group. The tissues of 3 mice in each group were sliced for observation. Data from at least three independent calculations are showed as mean ± SD (ns=not significant).

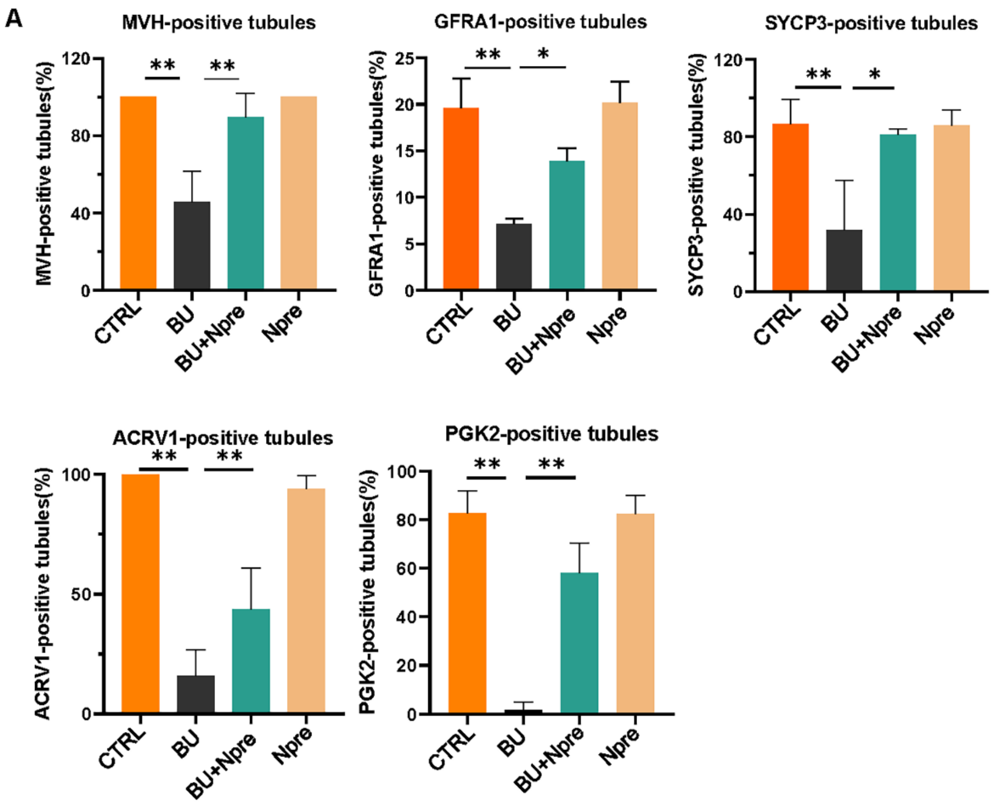

Figure S3. Effect of Npre supplementation on spermatogenesis in each group

(A) Percent of seminiferous tubules IF positive for the germ cell marker (MVH), spermatogonia stem cells (GFRA1), spermatocytes (SYCP3), spermatozoa (ACRV1) and high-quality sperm (PGK2) in testes of the indicated groups. In each group, about 160 tubules of four testes from 4 males were calculated. Data are shown as mean ± SD ($^{*}P < 0.05$; $^{**}P < 0.01$; ns=not significant).

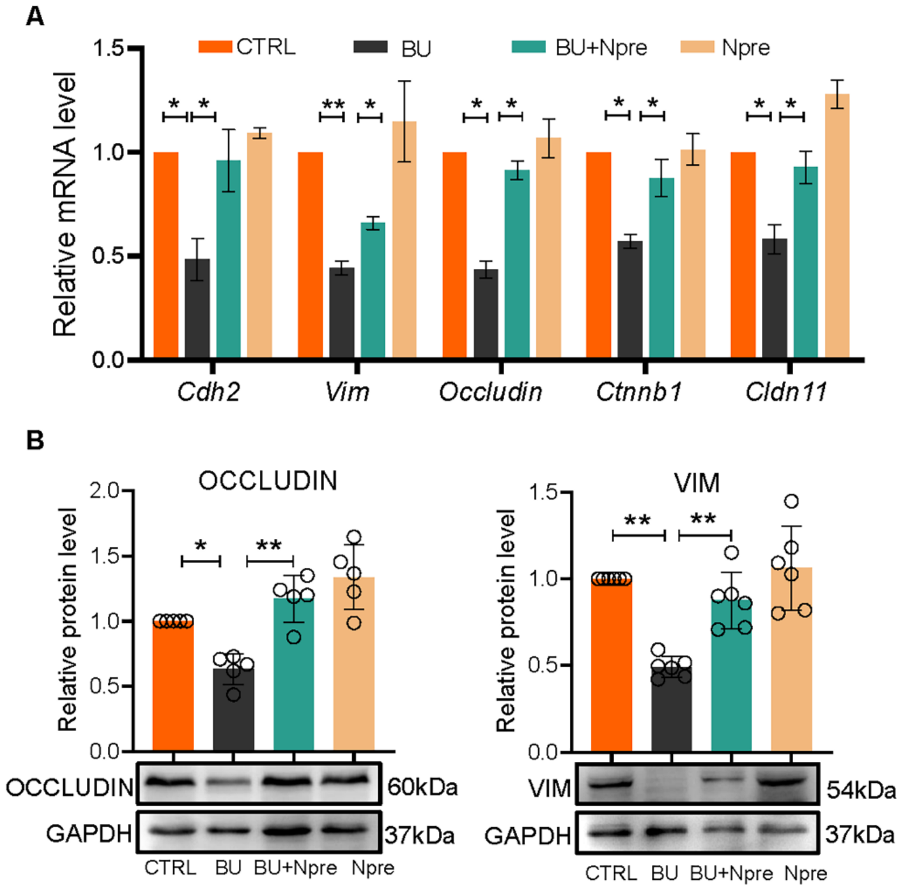

Figure S4. Npre supplementation alleviates the effect of BU on testicular BTB

(A) RT-qPCR for the mRNA levels of *Cdh2*, *Vim*, *Occludin*, *Ctnnb1* and *Cldn11* in each group. (B) WB images and the relative protein level of OCCLUDIN and VIM. (the levels of the proteins were calculated relatively to GAPDH). Testis samples were taken from at least 3 to 6 mice in each group. Data from at least three independent replicates are showed as mean ± SD (**P* < 0.05; ***P* < 0.01).

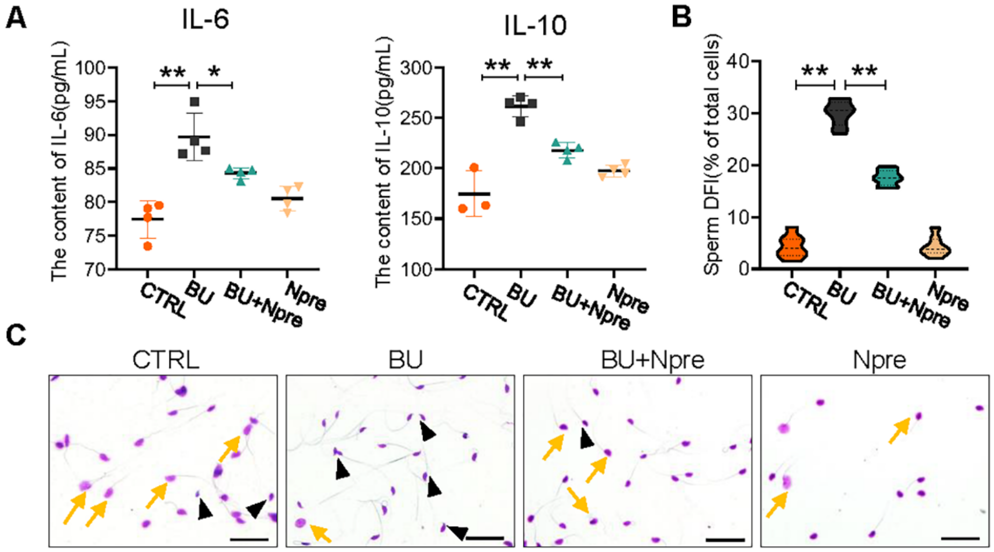

Figure S5. Pro-inflammatory cytokines levels and sperm DNA fragmentation index in epididymis

(A) Pro-inflammatory cytokine levels in the epididymis of the indicated groups. (B-C) DFI levels of sperm in each group. Scale bar = 20 μm. The sperm with halos of spreading of DNA loops indicated by the yellow arrow is normal sperm; The black arrowheads represent sperm without halos, which are sperm with DNA fragmentation. The content of pro-inflammatory cytokines in the epididymis of 3 male mice was determined. Data from at least three independent calculations are showed as mean ± SD ($^{*}P < 0.05$; $^{**}P < 0.01$, ns=not significant).

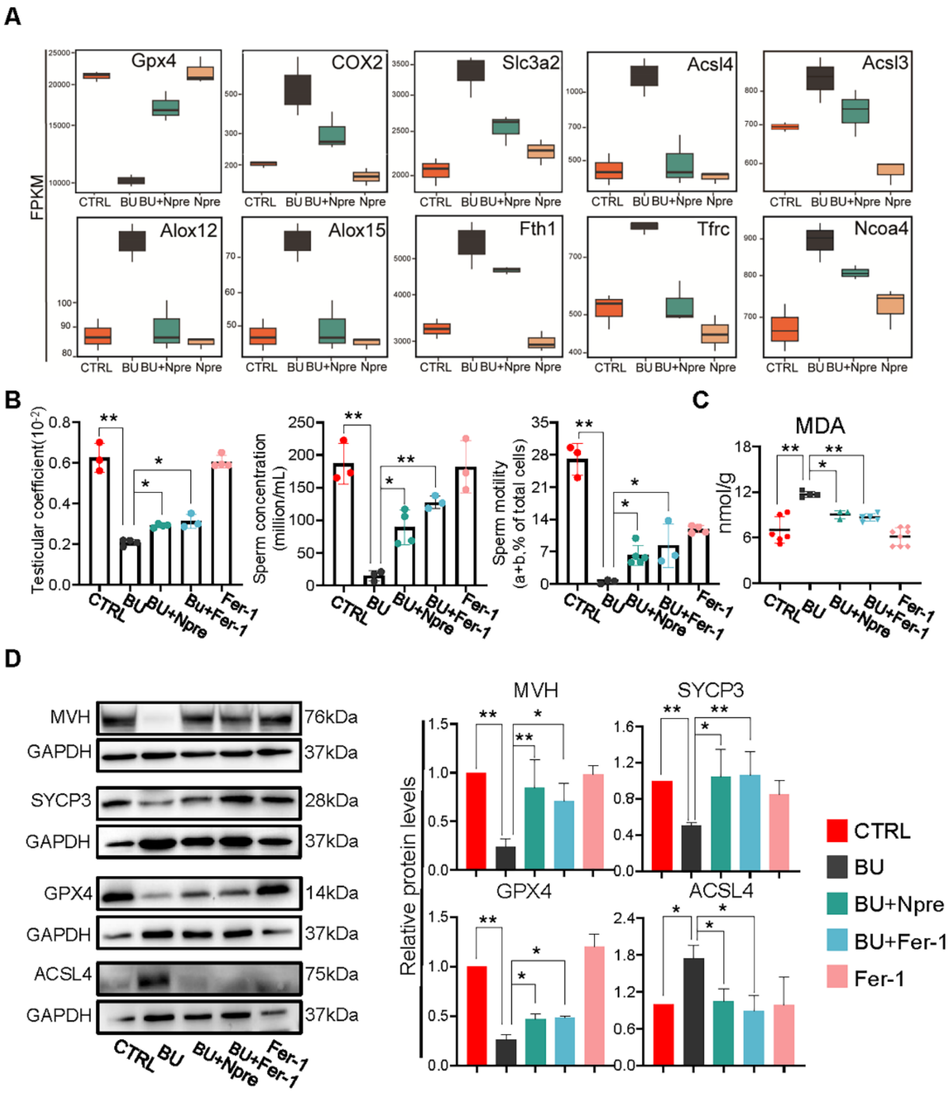

Figure S6. Inhibition ferroptosis in testis lessened spermatogenesis disorder caused by BU

(A) Expression level of ferroptosis gene markers calculated by FPKM method in the testes of the indicated groups. Comparison of the ameliorative effects of Npre and Fer-1 supplementation on testicular coefficient, sperm number and motility (B), and testicular concentration of the ferroptosis marker MDA (C) in BU treated males. (D) Left, representative WB of germ cell (MVH, SYCP3) and ferroptosis (GPX4, ACSL4) proteins; Right, quantification, relative to GAPDH, of the amount of these proteins in the testes of the indicated groups. Testis samples were taken from at least 3 to 6 mice in each group. Data from at least three independent replicates are showed as mean ± SD (*$P < 0.05$; **$P < 0.01$).

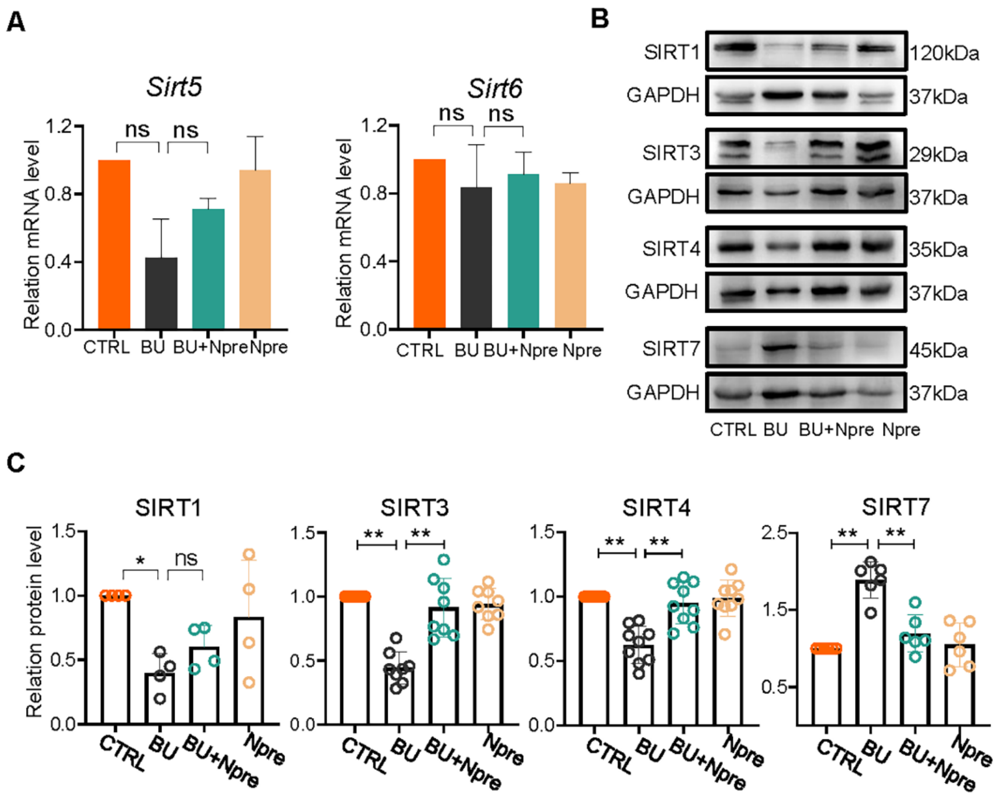

Figure S7 Npre supplementation alleviates the effect of BU on some members of sirtuin family

(A) RT-qPCR for *Sirt5* and *Sirt6* mRNA levels in each group. (B) Representative WB for SIRT1, 3, 4, 7 proteins and (C) quantitative evaluation of their expression in the four experimental groups (the levels of the proteins were calculated relatively to GAPDH). Testis samples were taken from at least 3 to 6 mice in each group. Data from at least three independent replicates are showed as mean ± SD (*$P < 0.05$; **$P < 0.01$, ns=not significant).

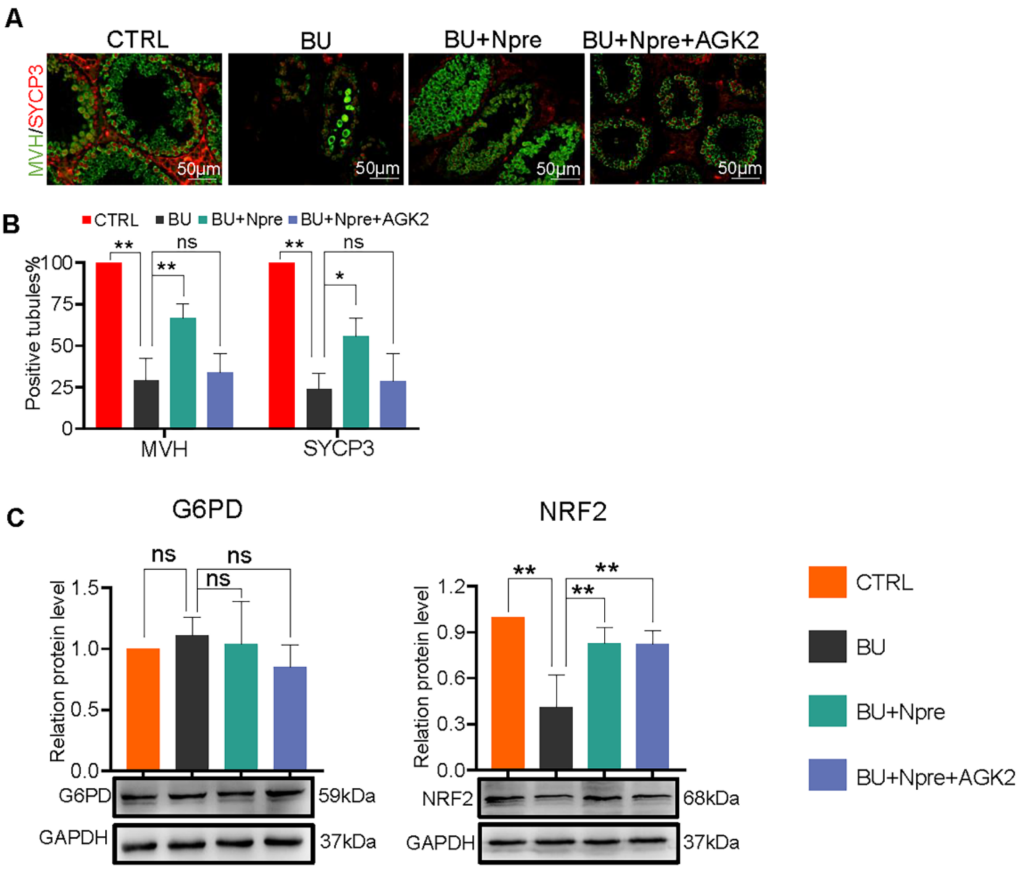

Figure S8. Effect of inhibition of SIRT2 on rescue effect of Npre

IF of seminiferous tubules for MVH and SYCP3 (A) and count of tubules positive for such proteins (B) showing significant reduction of the beneficial effect of Npre on the expression of both proteins in the testes of BU treated mice by AGK2. (C) Representative WB and quantification of the protein levels of G6PD and NRF2. Testis samples were taken from at least 3 to 6 mice in each group. Data from at least three independent replicates are showed as mean ± SD (**P* < 0.05; ***P* < 0.01; ns=not significant).

Table S1: The fertility statistics of each group.

Group	Number of co-cage female mice	Number of pregnancy mice	number of live pups/litter
CTRL	23	19	11.83±1.6
BU	25	1	5
BU+Npre	29	8	7±2.28
Npre	34	29	12.20±2.17

About 12-15 male mice were used to mate in each group
