# Supplementary material for: NAD+ precursors promote the restoration of spermatogenesis in busulfan-treated mice through inhibiting Sirt2-regulated ferroptosis
Source: Theranostics. 2024 Apr 15;14(6):2622–36. doi: 10.7150/thno.92416 (PMC11024856; doi:10.7150/thno.92416)
Supplement: Supplementary file 3 — Supplementary table 3. [file thnov14p2622s3.pdf]

List of primary antibodies

Antibody	Host	WB	IF/IHC	Supplier
DDX4	Rabbit	1:1000	1:200	Abcam (ab13840)
DDX4	Mouse	1:1000	1:200	Abcam (ab27591)
GFRA1	Rabbit	1:1000	1:200	Abclonal (A5373)
SYCP3	Mouse	1:1000	1:200	Abcam (ab97672)
ACRV1	Rabbit	1:1000	1:200	Proteintech (14040-1-AP)
PGK2	Rabbit	1:1000	1:200	BBI (D121903)
GAPDH	Rabbit	1:1000		Affinity (AF7021)
GPX4	Rabbit	1:1000	1:200	Abclonal (A1933)
ACSL4	Rabbit	1:1000		Abclonal (A6826)
COX2	Rabbit	1:1000		Abmart (T58852)
SIRT2	Rabbit	1:1000	1:50	Abclonal (A0237)

List of secondary antibodies

Antibody	WB	IF/IHC	Supplier
Goat anti-mouse lgG H&L (Alexa Fluor® 488)		1:200	Abcam (ab150113)
Goat anti-rabbit lgG H&L (Alexa Fluor® 488)		1:200	Abcam (ab150077)
Donkey anti-mouse lgG H&L (Alexa Fluor® 555)		1:200	Abcam (ab150106)
Donkey anti-rabbit lgG H&L (Alexa Fluor® 555)		1:200	Abcam (ab150074)
HRP-conjugated goat anti- Mouse IgG (WB)	1:2000	1:200	Beyotime (A0216)
HRP-conjugated goat anti- rabbit IgG (WB/IHC)	1:2000	1:50	Beyotime (A0258)
