# Supplementary material for: NAD+ precursors promote the restoration of spermatogenesis in busulfan-treated mice through inhibiting Sirt2-regulated ferroptosis
Source: Theranostics. 2024 Apr 15;14(6):2622–36. doi: 10.7150/thno.92416 (PMC11024856; doi:10.7150/thno.92416)
Supplement: Supplementary file 4 — Supplementary table 4. [file thnov14p2622s4.pdf]

List of primers

Primer	Sequence	Application
Gapdh-F	GTCATTGAGAGCAATGCCAG	qPCR
Gapdh-R	GTGTTGCTACCCCCAATGTG	qPCR
Mvh-F	TCAGACGCTCAACAGGATGT	qPCR
Mvh-R	ACTGGATTGGGAGCTTGTGA	qPCR
Gpx4-F	GCCAAAGTCCTAGGAAACGC	qPCR
Gpx4-R	CCGGGTTGAAAGGTTCAGGA	qPCR
Acsl4-F	AGCGTTCCTCCAAGTAGACC	qPCR
Acsl4-R	GTCCTTCGGTCCTAGTCCAG	qPCR
Sirt2-F	GCAGAACATAGACACGCTGG	qPCR
Sirt2-R	CCTGGGAGTTGCTTCTGAGA	qPCR
Sirt4-F	TCAATGTGCTGGATGGGTCT	qPCR
Sirt4-R	CAGAGACTCAAGACGCCAGA	qPCR
Sirt7-F	GCACTTGGTTGTCTACACGG	qPCR
Sirt7-R	ACAGTTCTGAGACACCACGT	qPCR
